# Sedative, hypnotic, anxiolytic and opioid medicament use and its co-occurrence with tobacco smoking and alcohol risk drinking in a community sample

**DOI:** 10.1186/1471-2458-7-337

**Published:** 2007-11-20

**Authors:** Ulrich John, Sebastian E Baumeister, Henry Völzke, Christian Meyer, Sabina Ulbricht, Dietrich Alte

**Affiliations:** 1 University of Greifswald, Institute of Epidemiology and Social Medicine, Walter-Rathenau-Str. 48, D-17487 Greifswald, Germany

## Abstract

**Background:**

Sedative, hypnotic, anxiolytic and opioid medicament (SO) use and its relation to tobacco smoking and alcohol risk drinking is largely unknown. Prevalence data for SO intake and its co-occurrence with tobacco smoking and alcohol risk drinking considering age are presented.

**Methods:**

Random general population sample of individuals aged 20–79 drawn from a mixed rural and urban area in Germany (Study of Health in Pomerania, SHIP). All medicament intake during the past 7 days prior to the interview was assessed according to the Anatomical Therapeutic Chemical classification as part of an interview conducted in a health examination center.

**Results:**

Among men, 3.0%, and among women 5.0% took SO. The proportion of SO users was higher (odds ratio 1.9; 95% confidence interval 1.1–3.4) whereas the proportions of current cigarette smokers and alcohol risk drinkers without SO use were lower among individuals aged 60–79 compared to those aged 20–39. The proportion of individuals with smoking, alcohol risk drinking or SO use was also lower among those aged 60–79 compared to the 20–39 year olds.

**Conclusion:**

Although proportions of SO users in older adult age are higher than in younger adult age there are less subjects with any of the 3 substance use behaviors at older adult age compared to age 20–39.

## Background

Little is known about (1) sedative, hypnotic, and anxiolytic (SHA), particularly benzodiazepine, and about (2) opioid medicament use behavior in general adult populations. Both substance groups, SHA and opioids taken together (SO) are related to health disorders, including dependence, abuse, intoxication, and withdrawal [1]. Benzodiazepines have been shown to be the most prevalent substance taken among mental health-related drugs in a general population sample aged 65 or older in Canada [2]. In France, a telephone survey study of the general population aged 18 or older revealed that 7.5% used benzodiazepines and altogether 11.5% used drugs against "anxiety, stress, to sleep or to relax [3]."

Generally, there are two alternative approaches to collect data on medicament use in general population studies. The first is to ask for medicament use for specific purposes, e. g. as a sleeping aid. The second approach involves asking for any medicament use irrespective of its purpose. Limitations of asking for medicament use by purpose are that (1) those consumers are not considered who take psychotropic medicines without having knowledge of the indication or effect of the substance and the potential dependence-related consequences of its intake and (2) consumers who might intentionally conceal non-medical use of substances. Asking for any medicament consumption or collecting medicament package data has the advantage to potentially reduce reporting bias, although this approach does not allow to separate non-medical from medical use.

Evidence is needed about the co-occurrence of SO use with tobacco smoking and alcohol risk drinking in the adult general population since data revealed higher morbidity risks for co-occurrent smoking and risk drinking than might be expected from the sum of risks of single substance use [4,5]. The common addictive nature of the substances makes it useful to describe whether there is co-occurrent use and to what extent and in which subgroups of the sample to a particular high degree. Co-use of these substances may be relevant for explaining common diseases. A co-occurrence might be explained by intentions to regulate body sensations such as upward and downward regulation of mood or use of different drugs for the same end. Alcohol and SO consumption might be interdependent because some of their effects, such as sedation, are similar [cf. [6]]. But SO consumption might be preferred because it is less obvious in the public. In the 1991 to 1993 US National Household Survey on Drug Abuse respondents with daily alcohol use had higher odds for prescription drug nonmedical use than individuals with less than daily alcohol use [7]. In a region of France, a postal survey of the general population aged 18 to 74 revealed that 28.6% smoked, 8.6% had excessive alcohol consumption, and 16.8% used medicaments for sleeping, against tiredness, against nervousness or anxiety [8]. In the US National Survey on Drug Use and Health more current smokers and more individuals with frequent binge drinking in the past two weeks were among benzodiazepine users than among non-users [9].

Contrary to SO use, tobacco smoking and alcohol risk drinking is less frequent in older than in younger adult age according to survey data, both among male and female users [10]. Data from Germany revealed that the proportion of current smokers among ever smokers is lower among older adult age groups than among younger adult age groups [11]. Older respondents in France included lower proportions of smokers but higher proportions of psychotropic medicament users [8]. Major shortcomings of this study included that medicaments were assessed with respect to their purpose; no data about the co-occurrence of different substance use behaviors were provided and that the survey had a response proportion of only 44%. Taken together, we lack evidence about SO use and its co-occurrence with tobacco smoking and alcohol risk drinking from general population samples that include young and older adult age.

The aim of this paper is to present, firstly, prevalence data about SO use stratified by gender, age, education, income, utilization of medical care, and a screening of psychiatric diagnoses. Secondly, the co-occurrence of SO use with current tobacco smoking and alcohol risk drinking will be analyzed. We hypothesized that (1) among smokers and alcohol risk drinkers, particularly among subjects who smoke and additionally drink alcohol in a risky way, more SO users may be found than among individuals who are nonsmokers and non-alcohol problem drinkers, and (2) in young and middle adult age there is a predominance of cigarette smoking and alcohol risk drinking whereas in older adult age SO use is more prevalent than current cigarette smoking and alcohol risk drinking. Accordingly, the use of any of the three substances is expected to be equally distributed over young, middle and older adult age.

## Methods

### Sample

The sample comprised all subjects from a random population sample drawn in a north-eastern German region. The sample is representative for the population of 212157 residents aged 20 – 79 in an area of West Pomerania in North-Eastern Germany. There were 7008 eligible individuals within this age range, stratified by 5-year strata and gender from residents' registration office files, in which every resident has to be registered by law, in three cities of 20,000 to 55,000 inhabitants and 29 surrounding towns and villages in West Pomerania. Of all eligible individuals, a total of 4310 (2193 females, 2117 males; response proportion: 68.8%), after having given written informed consent, took part in a health examination. It was carried out at the university hospital [12]. Among the participants, 20 had no or unfinished interview or incomplete data or refused to answer the questions for recent use of medication, leaving 4290 subjects as our final sample. The project was approved by the human research ethics committee of the Medical School at the University of Greifswald. Data were collected between 1997 and 2001. In the eastern German area in which the data were gathered there was no substantial illegal drug use known until 1990 due to the fact that the borders had been strictly controlled and there had been no purchasing power that might be interesting for an illegal drug market.

### Assessments

Medicament intake was assessed using the information of the drug packages if they were available. Otherwise the respondent provided information on the name of the specific medicament. The study aims and single parts of the health examination were explained to the individuals in brief in the invitation letter and in detail as a first step after having entered the health examination center. Subjects who had agreed to participate in the study were informed to bring the prescription sheets and the packages of all medications they had been taking during the past 7 days prior to the health examination. Subjects were asked as part of the interview: "During the last seven days: have you taken any medication such as tablets, drops, suppositories, or have you had any injection?" If this was the case, every compound was recorded. If available, the German central pharmaceutics number, a unique identification code, was recorded for linking it with the German drug databases. Compounds were identified and categorized using the Anatomical Therapeutic Chemical (ATC) classification [13] by local pharmacists who worked with two drug databases, which are widely used by pharmacists and scientists in Germany: the database (version Nov 30, 2001) of the Research Institute of the Health Insurance company AOK (WidO) and the database of the Federal Pharmacists' Association (ABDA, version Dec 1, 1999). Both databases together cover every compound that is licensed and available in Germany.

Due to the fact that not all participants brought their medicament packages with them and due to insufficient information provided by the respondent, not all compounds could be fully identified. When the pharmaceutics number was not provided the drug name was searched in the drug databases. The best fitting medicament was selected (called "proxy drug"), leading to a proxy pharmaceutics number. If no choice was possible for a specific drug only the ATC code was selected. Seventy two medicaments remained unidentified, mainly due to non-specific or false drug names given by the respondent. These medicaments were excluded from the analyses, not however the persons. Compounds with real or proxy pharmaceutical number could, in most of all cases, be linked directly to the ABDA database. If only the ATC but not the pharmaceutical number was available the ATC was used as a link.

SO were defined in accordance with SHA and licit opioids according to the Diagnostic and Statistical Manual of mental disorders (DSM-IV) [1]. The single medicaments were classified according to the ATC [13]. SHA included barbiturates, benzodiazepines or benzodiazepine-like substances, carbamates, and other sedatives, hypnotics or anxiolytics in accordance with DSM-IV. We used medicaments other than SO in our analysis, not however vitamin or mineral substituents, tonics (ATC groups A11, A12, A13) or hormonal contraceptives (ATC G03AA, G03AB, G03AC).

Cigarette smoking was assessed by interview questions. Current cigarette smokers were those who answered "Yes" to the question "Do you currently smoke cigarettes?". Alcohol consumption was assessed based on two interview questions: "How much beer, wine, and spirits did you drink at the last working day (but not Friday)?" and "How much beer, wine, and spirits did you drink at the last weekend, i. e. Saturday and Sunday?" We calculated grams pure alcohol by the multiplying volume % (4.8 for beer, 11.0% for wine, and 33.0% for spirits) with 0.794 and with liters of each drink. The product provided the grams pure alcohol. Risk drinking was defined as drinking more than 20 gr pure alcohol per day among women, and more than 30 gr pure alcohol per day among men [14]. School education, income, utilization of outpatient medical care including the specialization of the treating physician during the past 12 months and inpatient medical care including the number of days in hospital in the past 12 months was also assessed by computer-assisted personal interview. School education was divided into 3 groups of years of school education (<10, 10, > 10 years) according to the German school system. These 3 groups give information about the older and the younger study participants although the proportion of residents with 10 or more years school education has increased in Germany since the 1960ies. Income per month was estimated by income per household divided by the number of its members.

We screened for psychiatric disorders using the CID-S, a 12-item questionnaire for depressive, anxiety, somatoform, and substance use disorders during lifetime [15]. The following screening diagnoses were assessed: somatoform disorder (no, yes), number of anxiety disorders (0–5), number of depressive disorders (0–2), mania (no, yes), alcohol use disorders (no, yes), medication abuse (no, yes), illegal drug consumption (no, yes). The CID-S questions are taken from the Composite International Diagnostic Interview (CIDI) [16], German version [17]. The CID-S development study revealed that among individuals who affirmed at least one CID-S item, the sensitivity ranged between 79.0 and 95.2% for the single current disorders according to the Diagnostic and Statistical Manual (DSM-IV) [18] covered by the CIDI [15].

### Statistical analysis

The statistical analysis included SHA and opioid use. SO use in total was calculated by firstly assessing opioid intake. SHA use was assessed among all individuals without opioid intake. Other medicament users were defined as using neither SHA nor opioids but other medicaments. We defined the substance use pattern by separating five alternative groups of individuals: (1) current cigarette smokers without alcohol risk drinking and without SO use, (2) current cigarette smokers who were also alcohol risk drinkers but did not use SO, (3) alcohol risk drinkers who were neither current cigarette smokers nor SO users, (4) all SO users, (5) individuals who were neither current cigarette smokers nor alcohol risk drinkers nor SO users.

Proportions and multivariable regression models were estimated using SVY procedures in the Stata software package [19] as the complex sampling strategy in this study required adjustments in calculating the parameter estimates and standard errors. We calculated likelihood ratio chi^2 ^tests. According to medicament use, chi^2 ^tests were first calculated for differences among all individuals including users of SHA, opioids, other medicaments, and individuals who did not use any medicaments. Second, chi^2 ^tests were calculated to test for differences among the three subgroups of medicament users only (SHA, opoioids, other medicaments). We used the effect size measure Cohen's w [20] to analyze bivariable associations. According to Cohen [20], we interpreted values .10 to < .30 as indicating a small and .30 to < .50 a medium effect size. Odds ratios and 95% confidence intervals (CI) are reported for multinomial logistic regression and for binary logistic regression analysis. Age was grouped into young (20 to 39 years), middle (40 to 59 years), and older adult age (60 to 79 years) since national data from Germany had revealed that proportions of current smokers among male ever smokers were 64.1% at the age of 40–49 and 37.2% at age 60–69 compared to 95.5% among those younger than 20 years [11]. Further variables used for the data analysis were: school education (<10, 10, > 10 years), monthly income per household member (tertiles: < 950 €, 950 – < 1440 €, > 1440 €), outpatient care during the last 12 months prior to the health examination (neurologist or psychiatrist, GP but not neurologist or psychiatrist, other but neither neurologist, psychiatrist nor GP, no outpatient contact).

## Results

### Prevalence and determinants of SO use

Among the sample, 3.8% used SO, and 57.4% used medicaments other than SO. Among men, 3.0%, and among women 5.0% used one or more SO. There were 0.1% (6) barbiturate users, 2.5% (109) benzodiazepine users, 0.02% (1) carbamate user, 0.2% (7) users of other SHA, and 1.1% (49) opioid users. Among men, 3.0%, and among women 5.0% used SO (Table 1).

**Table 1:** Sedatives, hypnotics or anxiolytics (SHA), opioids, and other medicaments users

	Men					Women				
		
	SHA^a^	Opioids^b^	Other medicaments	No medicaments	Total	SHA^a^	Opioids^b^	Other medicaments	No medicaments	Total
										
Total	2.1	0.9	55.8	41.2	100.0	3.5	1.5	60.6	34.4	100.0
All individuals	Chi^2 ^27.5; df 3; p < 0.001; w .08									
All medicament users	Chi^2 ^6.6; df 2; p <.05; w .05									
Age (years)										
20–39	1.5	0.5	31.5	66.5	100.0	1.0	1.1	39.9	58.0	100.0
40–59	1.0	1.2	47.7	50.1	100.0	3.0	1.7	62.6	32.8	100.0
60–79	3.8	0.9	82.7	12.6	100.0	6.8	1.6	80.3	11.3	100.0
All individuals	Chi^2 ^490.5; df 6; p < 0.001; w .46					Chi^2 ^367.5; df 6; p < 0.001; w.40				
All medicament users	Chi^2 ^7.7; df 4; ns; w .08					Chi^2 ^13.7; df 4; p < 0.01; w .10				
Education (years)										
< 10	2.9	0.8	69.6	26.7	100.0	5.8	1.8	74.4	18.0	100.0
10	1.8	1.1	43.2	53.9	100.0	2.0	1.6	51.0	45.4	100.0
> 10	1.0	0.6	51.4	47.0	100.0	2.0	0.3	57.5	40.3	100.0
All individuals	Chi^2 ^149.5; df 6; p < 0.001; w .26					Chi^2 ^181.1; df 6; p < 0.001; w.28				
All medicament users	Chi^2 ^5.4; df 4; ns; w .07					Chi^2 ^13.4; df 4; p < 0.01; w .09				

Row per cent. Among the total sample, opioid users were identified. Among all non-opioid users, SHA users were identified.

Among all individuals who used neither opioids nor SHA, other medicament users were identified.

Chi^2^: Likelihood chi^2^-test, significance levels: p < 0.001, p < 0.01, p <  0.05, ns not significant.

df degrees of freedom.

w Cohen's w [20].

^a ^Sedatives, hypnotics, or anxiolytics: ATC groups N01AF, N01AG, N03AA, N05CA, N05CB, N03AE, N05BA, N05CD, N05CF, N05BC, N05CE, N05CM, N05CX [13] during the past 7 days prior to the health examination.

^b ^ATC groups A07DA52, N01AH, N02AA, N02AB, N02AC, N02AD, N02AE, N02AF, N02AG, N02AX, N02CX58 [13] during the past 7 days prior to the health examination.

SO use was not more prevalent among smokers or alcohol risk drinkers than among subjects who neither smoked nor drank alcohol in a risky way. Among male current cigarette smokers who also drank alcohol in a risky way, there were 2.3% and among female current cigarette smokers who also drank alcohol in a risky way there were 4.8% who also used SHA or opioids (Table 2). Smokers and risk drinkers were 1.6 to 3.7% among men and 4.7 to 5.1% among women. No associations were found between smoking or alcohol risk drinking and SO use: None of the smoker and risk drinker subgroups of the sample was different from subjects who had no medicament use as the reference group with respect to SO use in a multinomial logistic regression analysis after controlling for age and sex (Table 3)

**Table 2:** Sedatives, hypnotics or anxiolytics (SHA), opioids, and other medicaments users

	Men					Women				
		
	SHA^a^	Opioids^b^	Other medicaments	No medicaments	Total	SHA^a^	Opioids^b^	Other medicaments	No medicaments	Total
										
Total	2.1	0.9	55.8	41.2	100.0	3.5	1.5	60.6	34.4	100.0
Current smokers, alcohol risk drinkers										
Smoker, risk drinker	1.9	0.4	37.9	59.8	100.0	4.8	0.0	58.7	36.5	100.0
Smoker, non risk drinker	2.7	0.4	43.9	52.9	100.0	2.7	2.1	47.3	47.9	100.0
Non smoker, risk drinker	1.6	0.0	55.9	42.6	100.0	3.8	0.9	57.6	37.7	100.0
Non smoker, non risk drinker	2.2	1.5	65.1	31.2	100.0	3.7	1.4	65.6	29.3	100.0
All individuals	Chi^2 ^119.9; df 9; p < 0.001; w .24					Chi^2 ^65.0; df 9; p < 0.001; w .17				
All medicament users Age 20–39	Chi^2 ^11.9; df 6; ns; w .09					Chi^2 ^5.9; df 6; ns; w .06				
Current smokers, alcohol risk drinkers										
Smoker, risk drinker	2.4	0.8	30.4	66.4	100.0	0.0	0.0	50.0	50.0	100.0
Smoker, non risk drinker	2.1	0.5	35.2	62.2	100.0	1.1	1.8	38.6	58.5	100.0
Non smoker, risk drinker	1.2	0.0	29.4	69.4	100.0	0.0	2.9	38.2	58.8	100.0
Non smoker, non risk drinker	0.5	0.5	29.6	69.4	100.0	1.1	0.5	40.4	58.0	100.0
All individuals	Chi^2 ^6.1; df 9; ns; w .09					Chi^2 ^6.0; df 9; ns; w .09				
All medicament users Age 40–59	Chi^2 ^3.4; df 6; ns; w .12					Chi^2 ^5.3; df 6; ns; w .12				
Current smokers, alcohol risk drinkers										
Smoker, risk drinker	1.7	0.0	37.6	60.7	100.0	8.3	0.0	63.9	27.8	100.0
Smoker, non risk drinker	1.3	0.6	37.9	60.1	100.0	4.2	3.2	51.8	40.7	100.0
Non smoker, risk drinker	0.0	0.0	54.2	45.8	100.0	2.0	0.0	65.3	32.6	100.0
Non smoker, non risk drinker	1.0	2.5	53.3	43.2	100.0	2.2	1.5	66.0	30.3	100.0
All individuals	Chi^2 ^32.0; df 9; p < 0.001; w .20					Chi^2 ^18.8; df 9; p < 0.05; w .15				
All medicament users Age 60–79	Chi^2 ^13.4; df 6; p < 0.05; w .16					Chi^2 ^11.6; df 6; ns; w .15				
Current smokers, alcohol risk drinkers										
Smoker, risk drinker	0.0	0.0	74.1	25.9	100.0	0.0	0.0	100.0	0.0	100.0
Smoker, non risk drinker	6.2	0.0	70.8	22.9	100.0	5.0	0.0	73.3	21.7	100.0
Non smoker, risk drinker	4.3	0.0	82.8	12.9	100.0	13.0	0.0	69.6	17.4	100.0
Non smoker, non risk drinker	3.4	1.3	85.1	10.2	100.0	6.7	1.9	81.4	10.0	100.0
All individuals	Chi^2 ^22.8; df 9; p < 0.01; w .17					Chi^2 ^11.6; df 9; ns; w .13				
All medicament users	Chi^2 ^8.4; df 6; ns; w .10					Chi^2 ^4.3; df 6; ns; w .08				

Row per cent. Among the total sample, opioid users were identified. Among all non-opioid users, SHA users were identified.

Among all individuals who used neither opioids nor SHA, other medicament users were identified.

Chi^2^: Likelihood chi^2^-test, significance levels: p < 0.001, p < 0.01, p <  0.05, ns not significant.

df degrees of freedom.

w Cohen's w [20].

^a ^Sedatives, hypnotics, or anxiolytics: ATC groups N01AF, N01AG, N03AA, N05CA, N05CB, N03AE, N05BA, N05CD, N05CF, N05BC, N05CE, N05CM, N05CX [13] during the past 7 days prior to the health examination.

^b ^ATC groups A07DA52, N01AH, N02AA, N02AB, N02AC, N02AD, N02AE, N02AF, N02AG, N02AX, N02CX58 [13] during the past 7 days prior to the health examination.

**Table 3:** Associations between smoking and alcohol risk drinking with medicament use; multinomial logistic regression analysis

	Comparison group: no medicaments	
	
	SO^a ^use	Other medicament use
Smokers, risk drinkers		
Non smoker, non risk drinker	Ref	Ref
Smoker, non risk drinker	1.0 (0.8–1.4)	0.7 (0.6–0.8)
Non smoker, risk drinker	0.7 (0.4–1.0)	0.9 (0.7–1.2)
Smoker, risk drinker	1.0 (0.4–2.1)	0.7 (0.6–0.8)
Gender		
Men	Ref	Ref
Women	2.4 (1.6–3.5)	1.5 (1.3–1.8)
Age (years)		
20–39	Ref	Ref
40–59	2.7 (2.1–3.6)	2.3 (2.0–2.5)
60–79	18.3 (13.7–24.6)	11.1 (9.3–13.2)

Odds ratios (95%-confidence intervals).

Current smokers: current cigarette smokers, alcohol risk drinking according to [14],

^a ^Sedatives, hypnotics, or anxiolytics or opioids: ATC groups N01AF, N01AG, N03AA, N05CA, N05CB, N03AE, N05BA, N05CD, N05CF, N05BC, N05CE, N05CM, N05CX, A07DA52, N01AH, N02AA, N02AB, N02AC, N02AD, N02AE, N02AF, N02AG, N02AX, N02CX58 [13] during the past 7 days prior to the health examination.

Ref: Reference category.

In a further step, SO use was analyzed with respect to medical treatment. All SO users except one had contact to a physician during the past 12 months prior to the interview. Among individuals who had consulted a neurologist or a psychiatrist during the past 12 months, 10.6% took SHA and 3.4% took opioids (Table 4). Among those without a psychiatric screening diagnosis, less than 2% used SO compared to 8.2% among those with 3 or more psychiatric screening diagnoses.

**Table 4:** Sedatives, hypnotics or anxiolytics (SHA), opioids, and other medicaments users

	SHA^a^	Opioids^b^	Other medicaments	No medicaments	Total
					
Outpatient care 12 months					
Neurologist or psychiatrist	10.6	3.4	72.2	13.8	100.0
GP, not neurologist or psychiatrist	2.2	1.0	61.9	34.9	100.0
Other, not neurologist or psychiatrist	1.6	1.1	59.3	38.0	100.0
No	0.0	0.2	18.8	81.0	100.0
All individuals	Chi^2 ^557.9; df 6; p < 0.001; w .37				
All medicament users	Chi^2 ^66.7; df 6; p < 0.001; w .17				
Inpatient care (days)					
No	2.5	1.0	56.2	40.2	100.0
1–9	2.4	2.4	64.5	30.7	100.0
10 or more	5.9	2.2	73.7	18.2	100.0
All individuals	Chi^2 ^89.8; df 6; p < 0.001; w .14				
All medicament users	Chi^2 ^8.7; df 6; ns: w .06				
Psychiatric disorders (number)					
0	1.3	0.4	53.4	44.9	100.0
1 – 2	2.3	1.1	58.8	37.8	100.0
3 or more	5.8	2.4	64.3	27.6	100.0
All individuals	Chi^2 ^133.6; df 6; p < 0.001; w .18				
All medicament users	Chi^2 ^47.7; df 4; p < 0.001; w .14				

Row per cent. Among the total sample, opioid users were identified. Among all non-opioid users, SHA users were identified.

Among all individuals who used neither opioids nor SHA, other medicament users were identified.

Chi^2^: Likelihood chi^2^-test, significance levels: p < 0.001, ns not significant.

df degrees of freedom.

w Cohen's w [20].

^a ^Sedatives, hypnotics, or anxiolytics: ATC groups N01AF, N01AG, N03AA, N05CA, N05CB, N03AE, N05BA, N05CD, N05CF, N05BC, N05CE, N05CM, N05CX [13] during the past 7 days prior to the health examination.

^b ^ATC groups A07DA52, N01AH, N02AA, N02AB, N02AC, N02AD, N02AE, N02AF, N02AG, N02AX, N02CX58 [13] during the past 7 days prior to the health examination.

Women did not have higher odds for SHA or opioid use than men after adjustment for age, school education, income, outpatient care by a neurologist or psychiatrist, inpatient treatment, and psychiatric screening diagnoses, and using all subjects with use of other medicaments as the comparison group (Table 5). Since women and men did not differ according to the proportions of SHA or opioid users when the effect size measure Cohen's w was taken as the criterion we performed the multivariable data analysis without stratification for gender. Among individuals aged 60 to 79 higher odds were found for SHA intake when users of medicaments other than SO are the comparison group. School education of less than ten years was associated with higher odds for SHA intake than more than ten years school education when users of medicaments other than SHA or opioids were the comparison group. Individuals with 3 or more psychiatric diagnoses had higher odds for SHA or opioid intake compared to users of other medicaments.

**Table 5:** Associations between gender, age and sedative, hypnotic or anxiolytic and opioid medication use; multinomial logistic regression analysis

	Comparison group: no medicament use			Comparison group: other medicament use	
		
	SHA^a^	Opioids^b^	Other medicament	SHA^a^	Opioids^b^
					
Gender					
Men	Ref	Ref	Ref	Ref	Ref
Women	2.0 (1.3–2.9)	2.0 (1.1–3.8)	1.5 (1.3–1.7)	1.3 (0.9–1.9)	1.4 (0.8–2.4)
Age (years)					
20–39	Ref	Ref	Ref	Ref	Ref
40–59	2.5 (1.7–3.8)	2.6 (1.3–4.9)	2.3 (2.1–2.5)	1.1 (0.7–1.6)	1.1 (0.6–2.1)
60–79	25.7 (19.3–34.1)	11.5 (5.0–26.5)	10.9 (9.0–13.3)	2.3 (1.8–3.1)	1.0 (0.4–2.5)
Education (years)					
> 10	Ref	Ref	Ref	Ref	Ref
10	1.6 (0.99–2.6)	2.9 (1.2–7.4)	1.0 (0.8–1.2)	1.7 (1.0–2.7)	3.0 (1.2–7.8)
< 10	2.3 (1.5–3.6)	2.5 (0.9–6.7)	1.3 (1.0–1.5)	1.9 (1.2–3.0)	2.0 (0.7–5.8)
Income ^c ^(Euro)					
> 1440	Ref	Ref	Ref	Ref	Ref
950 – < 1440	0.7 (0.5–0.98)	0.8 (0.4–1.5)	0.8 (0.6–0.99)	0.9 (0.5–1.5)	1.0 (0.5–2.4)
< 950	1.0 (0.7–1.4)	1.1 (0.7–1.8)	0.8 (0.6–0.9)	1.4 (0.96–1.9)	1.5 (0.8–2.7)
Outpatient care					
not neurologist or psychiatrist	Ref	Ref	Ref	Ref	Ref
neurologist or psychiatrist	8.6 (4.5–16.4)	3.8 (2.5–5.7)	2.6 (2.0–3.3)	3.4 (2.0–5.7)	1.5 (0.97–2.3)
Inpatient care (days)					
no	Ref	Ref	Ref	Ref	Ref
1–9	1.0 (0.5–2.1)	3.3 (1.4–7.6)	1.5 (1.2–1.9)	0.7 (0.3–1.4)	2.2 (1.0–5.0)
10 or more	2.4 (1.4–4.2)	3.0 (1.5–5.9)	2.0 (1.5–2.7)	1.2 (0.7–1.9)	1.5 (0.9–2.7)
Psychiatric disorders, number					
0	Ref	Ref	Ref	Ref	Ref
1 – 2	2.3 (1.2–4.6)	2.4 (1.2–4.5)	1.4 (1.3–1.5)	1.7 (0.9–3.2)	1.7 (0.9–3.2)
3 or more	6.7 (3.5–13.0)	6.5 (4.2–9.9)	2.0 (1.7–2.3)	3.4 (1.8–6.5)	3.2 (2.1–5.0)

Odds ratios (95%-confidence intervals).

^a ^Sedatives, hypnotics, or anxiolytics: ATC groups N01AF, N01AG, N03AA, N05CA, N05CB, N03AE, N05BA, N05CD, N05CF, N05BC, N05CE, N05CM, N05CX [13] during the past 7 days prior to the health examination.

^b ^ATC groups A07DA52, N01AH, N02AA, N02AB, N02AC, N02AD, N02AE, N02AF, N02AG, N02AX, N02CX58 [13] during the past 7 days prior to the health examination.

^c ^Household-income divided by number of persons per household.

Ref: Reference category.

### Substance use pattern

Among men, 50.9% had one or more substance use risk behaviors, among women 35.3% (Table 6). Effect sizes were largest for the differences by age groups. Among men aged 60 to 79, there were 15.2% current cigarette smokers without SO use and 4.7% SO users in contrast to 50.8% current smokers without SO use and 2.0% SO users at age 20 to 39. Women aged 60 to 79 included 8.8% current cigarette smokers without SO use and 8.4% SO users in contrast to 41.5% current cigarette smokers without SO use and 2.1% SO users among women at age 20 to 39. According to the number of substance use behaviors among tobacco smoking, alcohol risk drinking, and SO use, 34.1% of the sample revealed one, and 8.9% two or three of the substance use risks. Among all men who practiced at least one of the 3 substance use behaviors, 6.0% used SO, and among women 14.1% used SO (Chi^2 ^223.1; df 3; p < 0.001; w .34). The data revealed that the proportions of individuals with at least one substance use risk behavior were lower in older than in younger age groups: among the young adults, 56.7%, among the middle adult age individuals, 46.8%, and in the older age group 26.3% disclosed at least one substance use behavior (Chi^2 ^281.0; df 2; p < 0.001; w .25).

**Table 6:** Substance use patterns

	Men						Women					
		
	Current smokers, no alcohol risk drinking, no SO^a ^use	Current smokers, alcohol risk drinking, no SO^a ^use	No current smokers, alcohol risk drinking, no SO^a ^use	All SO^a ^users	No substance use	Total	Current smokers, no alcohol risk drinking, no SO* use	Current smokers, alcohol risk drinking, no SO^a ^use	No current smokers, alcohol risk drinking, no SO^a ^use	All SO^a ^users	No substance use	Total
												
Total	20.3	12.5	15.1	3.0	49.1	100.0	22.9	2.8	4.6	5.0	64.7	100.0
	Chi^2 ^330.5; df 4; p < 0.001; w .27											
Age (years)												
20–39	30.9	19.9	13.8	2.0	33.5	100.0	37.8	3.7	4.6	2.1	51.8	100.0
40–59	20.6	15.8	19.8	2.2	41.7	100.0	21.6	4.1	5.9	4.7	63.7	100.0
60–79	11.7	3.5	11.6	4.7	68.4	100.0	8.6	0.2	3.0	8.4	79.8	100.0
	Chi^2 ^281.1; df 8; p < 0.001; w .36						Chi^2 ^249.2; df 8; p < 0.001; w .32					
Education (years)												
< 10	19.2	9.6	12.4	3.7	55.0	100.0	15.7	1.1	3.2	7.6	72.5	100.0
10	23.4	16.4	16.9	2.9	40.4	100.0	30.7	3.3	4.2	3.6	58.2	100.0
> 10	14.8	9.5	17.4	1.6	56.8	100.0	15.9	4.9	10.1	2.3	66.9	100.0
	Chi^2 ^64.2; df 8; p < 0.001; w .17						Chi^2 ^123.0; df 8; p < 0.001; w .24					
Income^b ^(Euro)												
< 950	25.3	17.3	13.8	3.3	40.3	100.0	30.1	4.8	2.8	4.5	57.8	100.0
950 – < 1440	20.1	11.0	16.0	2.6	50.3	100.0	22.2	1.4	5.4	4.4	66.6	100.0
> 1440	15.1	9.5	16.4	2.8	56.3	100.0	15.1	2.0	6.2	5.8	70.9	100.0
	Chi^2 ^56.9; df 8; p < 0.001; w .17						Chi^2 ^73.9; df 8; p < 0.001; w .19					
Outpatient care												
Neurologist or psychiatrist	14.0	6.0	13.5	14.0	52.4	100.0	15.3	2.4	3.5	13.9	64.8	100.0
GP, not neurologist or psychiatrist	20.4	11.8	15.1	2.0	50.7	100.0	22.9	2.7	4.3	4.4	65.7	100.0
Other, not neurologist or psychiatrist	15.9	10.7	17.8	3.7	51.8	100.0	21.5	2.4	7.2	1.7	67.2	100.0
No	28.1	21.1	13.4	0.3	37.1	100.0	41.3	4.9	4.9	0.0	49.0	100.0
	Chi^2 ^108.0; df 12; p < 0.001; w .26						Chi^2 ^103.0; df 12; p < 0.001; w .22					
Inpatient care (days)												
no	20.9	12.8	15.5	2.6	48.3	100.0	23.4	2.8	4.9	4.5	64.4	100.0
1–9	20.4	13.4	14.1	2.8	49.3	100.0	23.0	4.0	3.4	6.8	62.8	100.0
10 or more	15.5	9.3	11.9	7.2	56.2	100.0	17.7	1.2	2.4	9.2	69.5	100.0
	Chi^2 ^18.1; df 8; p < 0.05; w .10						Chi^2 ^14.9; df 8; ns; w .08					
Psychiatric disorders (number)												
0	19.9	12.9	16.2	0.9	50.1	100.0	21.9	2.2	4.1	2.8	69.0	100.0
1 – 2	21.7	11.7	14.4	2.8	49.4	100.0	26.4	1.5	4.6	4.0	63.6	100.0
3 or more	18.7	13.1	13.8	7.9	46.5	100.0	20.0	4.7	5.1	8.3	61.9	100.0
	Chi^2 ^46.8; df 8; p < 0.001; w .16						Chi^2 ^47.4; df 8; p < 0.001; w .15					

Row percent.

Chi^2^: Likelihood chi^2^-test, significance levels: p < 0.001, p < 0.05, ns  not significant.

df degrees of freedom.

w Cohen's w [20].

Current smokers: current cigarette smokers, alcohol risk drinking according to [14],

^a ^Sedatives, hypnotics, or anxiolytics or opioids: ATC groups N01AF, N01AG, N03AA, N05CA, N05CB, N03AE, N05BA, N05CD, N05CF, N05BC, N05CE, N05CM, N05CX, A07DA52, N01AH, N02AA, N02AB, N02AC, N02AD, N02AE, N02AF, N02AG, N02AX, N02CX58 [13] during the past 7 days prior to the health examination.

^b ^Household-income divided by number of persons per household.

Women revealed lower odds than men for substance use patterns without SO use, not however for SO use after adjustment for age, school education, income, outpatient care rendered by a neurologist or psychiatrist, inpatient treatment, and psychiatric screening diagnoses (Table 7). Therefore, we conducted further analysis without stratification for gender. Those aged 60 to 79 had an OR of 1.9 (CI 1.1–3.4) for SO use in contrast to lower odds for current smokers or alcohol risk drinkers compared to individuals who were neither current cigarette smokers nor alcohol risk drinkers nor SO users. Opposite findings for all SO users on the one hand side and smokers without SO use on the other hand side were also revealed for outpatient care provided by a neurologist or psychiatrist. Those with the lowest education had higher odds for smoking and for SO use compared to those who did not use any of the three substances. Substance use in total, current cigarette smoking, alcohol risk drinking or SO use, was lower among individuals at age of 40 or above compared to individuals younger than 40 years.

**Table 7:** Associations with substance use patterns; multinomial logistic regression analysis

	Comparison group: None of the 3 substances taken				Any substance use^a^
		
	Current smokers, no alcohol risk drinking, no SO^b ^use	Current smokers, alcohol risk drinking no SO^b ^use	No current smokers, alcohol risk drinking no SO^b ^use	All SO^b ^users^c^	
Gender					
Men	Ref	Ref	Ref	Ref	Ref
Women	0.7 (0.6–0.8)	0.13 (0.10–0.18)	0.2 (0.16–0.26)	1.1 (0.8–1.5)	0.4 (0.39–0.5)
Age (years)					
20–39	Ref	Ref	Ref	Ref	Ref
40–59	0.5 (0.4–0.6)	0.7 (0.56–0.99)	1.1 (0.9–1.5)	1.2 (0.7–2.1)	0.6 (0.5–0.8)
60–79	0.14 (0.10–0.18)	0.09 (0.06–0.15)	0.5 (0.3–0.6)	1.9 (1.1–3.4)	0.2 (0.17–0.3)
Education (years)					
> 10	Ref	Ref	Ref	Ref	Ref
10	1.9 (1.5–2.5)	1.5 (1.0–2.1)	0.9 (0.7–1.2)	2.2 (1.2–4.2)	1.5 (1.3–1.7)
< 10	2.3 (1.7–3.1)	1.5 (1.0–2.3)	0.8 (0.5–1.1)	2.3 (1.2–4.4)	1.5 (1.3–1.8)
Income^d ^(Euro)					
> 1440	Ref	Ref	Ref	Ref	Ref
950 – < 1440	1.3 (1.0–1.6)	1.1 (0.8–1.5)	1.1 (0.8–1.4)	0.9 (0.6–1.4)	1.1 (0.96–1.3)
< 950	1.4 (1.2–1.8)	1.6 (1.2–2.2)	0.9 (0.6–1.1)	1.4 (0.9–2.1)	1.3 (1.1–1.4)
Outpatient care					
not neurologist or psychiatrist	Ref	Ref	Ref	Ref	Ref
neurologist or psychiatrist	0.7 (0.5–0.996)	0.5 (0.3–0.9)	0.9 (0.6–1.3)	2.9 (2.0–4.2)	1.0 (0.9–1.2)
Inpatient care (days)					
No	Ref	Ref	Ref	Ref	Ref
1–9	1.0 (0.7–1.4)	1.3 (0.8–2.1)	1.0 (0.6–1.5)	1.2 (0.6–2.2)	1.0 (0.8–1.3)
10 or more	0.9 (0.6–1.3)	1.0 (0.6–1.7)	0.8 (0.5–1.3)	1.4 (0.9–2.2)	1.0 (0.8–1.2)
Psychiatric disorders (number)					
0	Ref	Ref	Ref	Ref	Ref
1 – 2	1.3 (1.1–1.5)	0.9 (0.6–1.2)	0.9 (0.7–1.2)	2.0 (1.2–3.3)	1.1 (0.8–1.7)
3 or more	1.1 (0.9–1.4)	1.4 (1.0–2.0)	1.0 (0.8–1.4)	4.2 (2.5–6.8)	1.3 (0.99–1.7)

^a ^Logistic regression analysis for any of the 3 substances used versus none of the 3 substances used.

Odds ratios (95%-confidence intervals).

Current smokers: current cigarette smokers, alcohol risk drinking according to [14],

^b ^Sedatives, hypnotics, or anxiolytics or opioids: ATC groups N01AF, N01AG, N03AA, N05CA, N05CB, N03AE, N05BA, N05CD, N05CF, N05BC, N05CE, N05CM, N05CX, A07DA52, N01AH, N02AA, N02AB, N02AC, N02AD, N02AE, N02AF, N02AG, N02AX, N02CX58 [13] during the past 7 days prior to the health examination.

^c ^This group includes SO users with or without current smoking or alcohol risk drinking. All subgroups of SO users were taken together because of small cell frequencies.

^d ^Household-income divided by number of persons per household.

Ref: Reference category.

### Discussion

There are four main findings. First, when taking SO use, tobacco smoking and alcohol risk drinking together, the substance-related health risk of the population is high throughout all age groups and in both genders. Second, the data suggest that there is no higher odds ratio for SO use among smokers and among alcohol risk drinkers compared to those who neither smoke nor drink alcohol in a risky way. Third, the data confirm findings from previous studies that show particularly high proportions of SO users at older age groups. Fourth, the proportion of SO users is lower than the proportion of current cigarette smokers or alcohol risk drinkers when all age groups are considered. The hypothesis is supported that in older adult age more SO use is present than in younger age groups, among men and among women. However, subjects with intake of any of the 3 substances were unequally distributed across younger, middle, and older adult age.

We did not find an association between current smoking or alcohol risk drinking with SHA or opioid use, neither in the total sample nor in single age groups. This finding indicates that there is no such relation with psychotropic medicine use in this sample as is known from smoking and risk drinking and the respective hypothesis is rejected. One explanation might be that SHA and opioid use were by far more rare than smoking in this sample. Easy availability of cigarettes and alcohol might add to that. On the other hand, particularly for elderly women a preference for SHA might be expected. However, there was no strong evidence for this.

The proportion of individuals who showed one or more risk behaviors among tobacco smoking, alcohol risk drinking and SO use is tremendously high among those younger than 40 with 66.5% among men and 48.2% among women. Substance use in older age is a prevalent health risk behavior, although the proportion of substance users at age 60 to 79 is less than half the respective proportion at age 20 to 39, both in women and men. We do not know the specific reasons for this difference. Selective mortality, smoking cessation and changes in alcohol consumption may have caused this finding.

The proportions of SHA or opioid users seem to be lower than those found in other research [3]. This might be due to differences in data collection methods or characteristics of the samples, such as age ranges. Furthermore, our data do not confirm that the group of benzodiazepine users contains more current smokers or alcohol risk drinkers than non-users [9]. The data confirm findings of a higher proportion of SO users in older than in young adult age [3,8] and they compare with data that revealed high proportions of benzodiazepine consumers among the elderly [2]. However, no differences by age were present when SO users are compared to individuals with other medicament intake except for SHA at age 60 to 79. The adjusted OR for SO users compared to individuals without smoking, without alcohol risk drinking and without SO use is 1.9 with a lower confidence bound close to 1 (CI 1.1 to 3.4). The bivariable statistics revealed higher proportions of individuals with SHA or opioid use in higher age groups among all medicament users only for women. Even in older adult age, smoking and alcohol risk drinking are the main substance use risk behaviors among men whereas among women the proportion with SO use is close to the proportion of current smokers and alcohol risk drinkers.

Less educated individuals and those with the lowest income had higher odds for current cigarette smoking and for SO use after adjustment compared to those with the highest school education and the highest income respectively. The data suggest that proportions of SO users are higher among those with a larger number of psychiatric disorders, not however the proportions of individuals with other SP.

Substance use patterns are different with respect to gender, age, education, and utilization of outpatient medical care. Women have substantially less alcohol risk drinking whereas they do not differ from men according to SO use. While there are less current smokers, with or without co-occurrent alcohol risk drinking, there are more SO users at age 60 – 79 compared to those younger than 40. Several explanations might hold for this finding. On the one hand side concerns about one's health status might be responsible for quitting smoking and alcohol risk drinking among the older individuals, whereas on the other hand problems of quality of life at older age, such as how to cope with sleeplessness, might stimulate SO use. However, there are differences by education and income indicating that individuals with less education presented higher proportions of smokers, alcohol risk drinkers and SO users. One reason might be that substance use and morbidity is higher in subpopulations with low compared to those with high socioeconomic status. One subgroup among SO users might be those who are in neurological or psychiatric outpatient treatment.

Our data do not allow conclusions according to non-medical or medical SO use. The data revealed more SO use among individuals who had consulted a neurologist or a psychiatrist during the past 12 months prior to the health examination than among individuals without service use. However, even among individuals in medical treatment, there may have been non-medical use if they took the substances in larger amounts than prescribed. Moreover, we could not identify the reasons for taking the substance including use according to prescription only, abuse or dependence on prescription drugs in conjunction with illicit drug use or prescription drug use when illicit drugs of choice were not available [21].

One objection against our measurement of substance use patterns is that current smoking, alcohol risk drinking and SO use may be comparable only in part. Differences in availability of the substances must be considered. Neither exists a standardization of the amount of toxic effects which the individuals are exposed to across tobacco smoke, alcohol and SO nor is there evidence of a standard score according to their risk potential. Diseases caused by tobacco smoking and alcohol risk drinking are largely known, and there also exists evidence about dose-response-relations. We know less from SO use. Our data did not include how long and in which dose SO have been taken. Guidelines for risks are available for alcohol consumption [e.g. [14]] whereas for SO use it seems to be largely unknown which lower limit exists for attributable morbidity and mortality depending on the single drug. There is some knowledge about the risk increase by the co-occurrence of tobacco smoking and alcohol risk drinking but we hardly know anything about how SO use interacts with consumption of the other two substances. Furthermore, tolerance and total lifetime doses of exposure until manifestation of disease that is attributable to the substance use may differ between men and women.

Further limitations of our study are: (1) The sample was drawn in one region of Germany only. It is not representative for the whole country due to its low population density and different social structure. However, we assume that this does not change the associations between smoking alcohol risk drinking and potential confounders with SO use as analyzed in this study. (2) No data about current illicit drug use were available. (3) The sample includes only individuals who were younger than 80 years when the sample was drawn. Above that age there seem to be less current smokers and alcohol risk drinkers whereas SO use may be more prevalent than in the age range of our sample [cf. [3,11,22]]. (4) In several cases the cell numbers were small. Thus, more significant differences may be expected in a larger sample for SHA and for opioid use for age, education and income, outpatient and inpatient care, and number of psychiatric disorders (5) This is a cross-sectional study. Thus, age cohort effects cannot be excluded. (6) For each of the three substance use behaviors different reporting bias may have been active, which is expected high for alcohol drinking amounts and low for smoking and SO use. The reason is that there were low health policy efforts to combat smoking and SO use at the time of the data collection or before that time. A strength of the present study is the data gathering of medicaments. This might have added to the reduction of reporting bias. But we may have missed some drugs. Recall bias may have been active among those who did not bring their medicament packages. Even among those who brought their drugs, some may have missed single packages. Individuals who had prescriptions actually may have not have taken the medication. (7) We do not know the duration of medicament intake. (8) Beyond drugs, the data have been gathered on grounds of self-statements and were not verified biochemically. However, other evidence suggests that in population-based studies, there is no considerable risk of denial which significantly influences the results [23].

## Conclusion

SO use is less prevalent than tobacco smoking or alcohol risk drinking in this general population sample. Although the proportion of individuals with SO use was higher in older than in younger adult age, any substance use among current cigarette smoking, alcohol risk drinking or SO use, was lower in older adult age than at age 20–39.

## Competing interests

The author(s) declare that they have no competing interests.

## Authors' contributions

UJ carried out the major part of the data analysis and wrote major parts of the first draft of the paper. SB carried out parts of the data analysis and assisted in further parts of the data analysis. HV and DA obtained the data, contributed parts of the text to the paper and assisted with the writing of the manuscript and in the interpretation of the results. CM and SU contributed ideas for the data analysis, assisted with the writing of the manuscript and in the interpretation of the results.

## Pre-publication history

The pre-publication history for this paper can be accessed here:


